# OCT in Oncology and Precision Medicine: From Nanoparticles to Advanced Technologies and AI

**DOI:** 10.3390/bioengineering12060650

**Published:** 2025-06-13

**Authors:** Sanam Daneshpour Moghadam, Bogdan Maris, Ali Mokhtari, Claudia Daffara, Paolo Fiorini

**Affiliations:** 1 Department of Engineering for Innovation Medicine, University of Verona, 37134 Verona, Italy; sanam.daneshpourmoghadam@univr.it (S.D.M.); paolo.fiorini@univr.it (P.F.); 2 Department of Computer Science, University of Verona, 37134 Verona, Italy; ali.mokhtari@univr.it (A.M.); claudia.daffara@univr.it (C.D.)

**Keywords:** optical coherence tomography (OCT), nanoparticles, artificial intelligence (AI), targeted imaging and precision medicine

## Abstract

Optical Coherence Tomography (OCT) is a relatively new medical imaging device that provides high-resolution and real-time visualization of biological tissues. Initially designed for ophthalmology, OCT is now being applied in other types of pathologies, like cancer diagnosis. This review highlights its impact on disease diagnosis, biopsy guidance, and treatment monitoring. Despite its advantages, OCT has limitations, particularly in tissue penetration and differentiating between malignant and benign lesions. To overcome these challenges, the integration of nanoparticles has emerged as a transformative approach, which significantly enhances contrast and tumor vascularization at the molecular level. Gold and superparamagnetic iron oxide nanoparticles, for instance, have demonstrated great potential in increasing OCT’s diagnostic accuracy through enhanced optical scattering and targeted biomarker detection. Beyond these innovations, integrating OCT with multimodal imaging methods, including magnetic resonance imaging (MRI), positron emission tomography (PET), and ultrasound, offers a more comprehensive approach to disease assessment, particularly in oncology. Additionally, advances in artificial intelligence (AI) and biosensors have further expanded OCT’s capabilities, enabling real-time tumor characterization and optimizing surgical precision. However, despite these advancements, clinical adoption still faces several hurdles. Issues related to nanoparticle biocompatibility, regulatory approvals, and standardization need to be addressed. Moving forward, research should focus on refining nanoparticle technology, improving AI-driven image analysis, and ensuring broader accessibility to OCT-guided diagnostics. By tackling these challenges, OCT could become an essential tool in precision medicine, facilitating early disease detection, real-time monitoring, and personalized treatment for improved patient outcomes.

## 1. Introduction and Background

Optical Coherence Tomography (OCT), first developed in 1991 by Huang et al., marked a revolutionary milestone in medical imaging technology [1]. Originally applied in ophthalmology, OCT provided high-sensitivity micrometer resolution and cross-sectional retinal imaging in vivo [2,3], significantly improving early detection and quantitative monitoring of a variety of retinal disorders [4]. Over the last decades, OCT has expanded beyond ophthalmology, becoming a useful diagnostic tool in dermatology, oncology, and other medical fields. OCT is a non-invasive, non-contact imaging technique based on low-coherence interferometry to create 3D volumetric images of biological tissues [5,6,7]. The underlying principle is a Michelson-type interferometer employing a low-coherence light source: the light beam is split into two paths, one directed toward the sample and the other to a reference mirror; the recombination of the reflected beams generates an interference pattern only when the two optical path lengths match within the short coherence length of the source, which is in the order of micrometers, thus enabling depth-resolved imaging. OCT technology has continually improved [8]. In early OCT devices, called time-domain OCT (TD-OCT), the depth profile of the sample was retrieved by varying the path of the reference mirror. More efficient implementation allowing fast acquisition was introduced by the second generation of OCT devices, the spectral-domain OCT (SD-OCT) [9], in which the depth information is extracted in one shot, from the interference spectrum. The image quality, high speed, and high sensitivity of SD-OCT, which became the standard, opened the way to volumetric imaging in vivo [10]. Advancements in laser technology led to the swept-source OCT [SS-OCT], a spectral-domain configuration in which a wavelength-swept laser is used for rapidly tuning the emitted wavelength over a broad range [11,12]. The axial resolution of OCT, determined by the coherence length of the light source, is typically in the order of 10 μm. This micrometer-scale precision is critical for resolving thin structures like retinal layers or dermal microstructures [13,14]. OCT’s imaging depth generally spans a few millimeters, which is sufficient for examining semi-transparent tissues such as the retina, although deeper penetration remains challenging for denser tissues [15]. By acquiring depth profiles [A-scans] in raster scanning, the OCT systems generate cross-sectional images (B-scans) and en face visualizations (C-scans), enabling comprehensive 3D imaging [16]. Lateral resolution, determined by the spot size of the probing beam, is limited by the optics.

A defining characteristic of OCT is its reliance on low-coherence light, which allows for precise measurement of optical path length differences based on interferometric detection of backscattered light from tissue layers. This enables the high axial resolution that sets OCT apart from imaging modalities like ultrasound or MRI [17,18]. However, the coherent nature of the light source inherently introduces challenges, such as speckle noise, which degrades image quality and makes fine structural details difficult to resolve. Recent advancements, including photonic lanterns, have addressed this issue by reducing speckle contrast and enhancing image quality [17,19]. Compared to other imaging techniques, OCT provides unique advantages. Ultrasound, which operates using sound waves, typically offers a resolution of around 200 μm, while MRI delivers excellent soft-tissue contrast but with lower spatial resolution. Moreover, OCT does not require ionizing radiation, making it safer for repeated use [20,21]. The performance of OCT depends on the properties of the light source. The spectral bandwidth of the OCT light source is indirectly proportional to the depth resolution. Superluminescent diodes (SLDs) are widely used for their broad spectral bandwidth and high output power, providing the micrometer-scale resolution required for clinical diagnostics [22]. Supercontinuum light sources, emerging as alternatives, offer ultra-broad spectral outputs for exceptional resolution and deeper tissue imaging, but their complexity and cost have limited widespread adoption [23]. Additionally, tunable lasers, often employed in frequency-domain OCT, enhance spectral data acquisition, enabling precise imaging of specific tissue types [24]. Performance parameters that are coupled are axial resolution and imaging depth. Shorter wavelengths provide higher resolution imaging but are limited by scattering in tissue, while longer wavelengths penetrate deeper into tissues but sacrifice resolution [25]. To overcome these trade-offs, innovations such as line-field confocal OCT (LC-OCT) and dual-channel systems have been developed [12], enhancing both imaging depth and resolution for broader clinical applications [26].

After recalling the background on the OCT technique, this review focuses on OCT’s transformative role in pathology, emphasizing its ability to visualize microstructures for diagnostics across ophthalmology, dermatology, oncology, and other medical applications. OCT’s non-invasive, real-time imaging capabilities have the potential for early detection and surgical guidance, offering significant advantages in patient care [27,28]. Recent advancements, including the integration of nanoparticles as contrast agents and multimodal imaging with MRI, demonstrate the growing potential of OCT in complex diagnostic workflows.

By bridging current clinical applications with emerging innovations, this review provides a comprehensive perspective on OCT’s evolution and its expanding role in modern medicine. The discussion of its technical foundations, limitations, and future possibilities aims to deepen understanding and highlight pathways for continued advancements in medical diagnostics.

## 2. Basics of OCT Imaging in Pathology

### 2.1. OCT’s Role in Pathological Diagnosis

As mentioned, OCT has emerged as a valuable high-resolution imaging tool in pathology, offering real-time cross-sectional visualization of biological tissues. Its non-invasive nature and ability to assess tissue microstructure make it a promising adjunct to conventional histopathological methods in diagnosing various diseases, including oncological, ophthalmological, neurological, and vascular disorders [1,29]. This section explores the role of OCT in detecting pathological changes, highlights the key histopathological features identifiable with OCT, and discusses its advantages and limitations.

**Figure 1:**
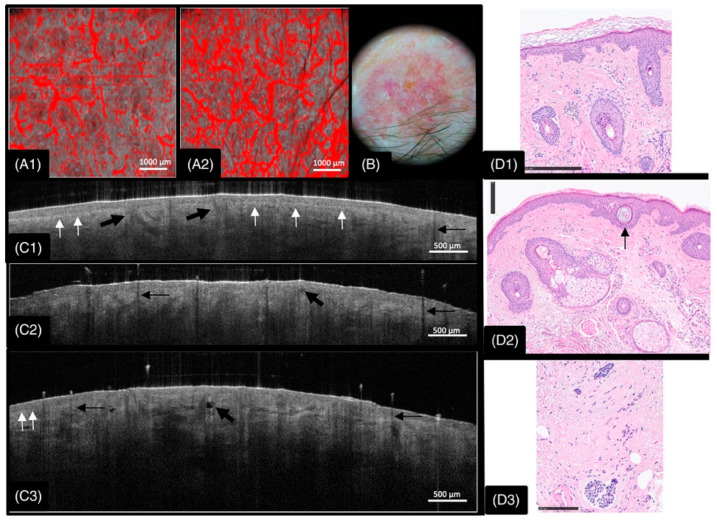
Imaging of morpheaform basal cell carcinoma (BCC) on the forehead above the eyebrow (Case 2), using dynamic and structural OCT alongside dermoscopy and histopathology. (**A1**) D-OCT of adjacent normal skin shows evenly distributed linear, dotted, and branching vessels. (**A2**) D-OCT of the central tumor region reveals increased vessel density and diameter, with serpiginous and branching patterns. (**B**) Dermoscopy image showing the clinical appearance of the lesion. (**C1**) Structural OCT of normal skin displays an intact dermoepidermal junction (DEJ) and hair follicles with shadowing. (**C2**,**C3**) OCT images near tumor borders (proximal and distal) show disrupted layering and partial DEJ loss, indicating tumor extension. Black arrows mark regions of architectural disruption and tumor nests; white arrows indicate areas of preserved layering and an intact DEJ. (**D1**,**D2**) Histopathology at distal (**D3**) and proximal (P3) scan sites reveals no visible tumor, in contrast to OCT-detected abnormalities. (**D3**) Histology from the tumor center confirms BCC with infiltrative basaloid strands [31].

OCT enables the detection of early structural abnormalities, which may relate to changes in tissue composition, extracellular matrix organization, and cellular density (Figure 1) [4,30]. Clearly, being an interferometry technique, OCT provides information on the structure of the sample, without any information on the material reflectance properties, like microscopy intensity-based imaging techniques.

**Figure 2:**
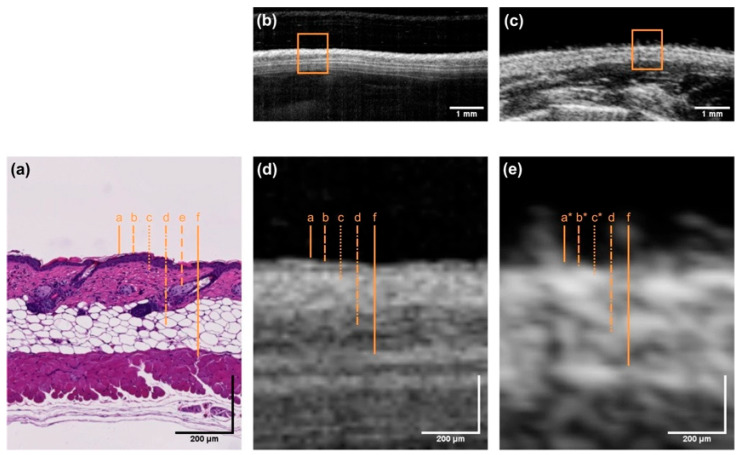
Exemplary cross-sectional images of murine skin were obtained by (**b**) OCT and (**c**) HFUS and corresponding (**a**) histology (H&E staining). Enlarged cross-sections, as indicated by the orange rectangles, are depicted in (**d**,**e**). Dashed orange lines in images (**a**,**d**,**e**) indicate the skin layers corresponding to (a) epidermis, (b) papillary dermis, (c) reticular dermis, (d) subcutis, (e) sebaceous glands and hair follicles, and (f) muscle. Skin layers with letters labeled with an asterisk are not distinguishable [32].

While histopathology remains the gold standard for diagnosing cellular-level abnormalities, OCT offers several advantages in real-time tissue assessment, non-invasive imaging, immediate diagnosis [7], dynamic imaging capabilities [16], and repeated monitoring (Figure 2) [29].

Multiple studies have quantitatively assessed OCT’s diagnostic performance compared to histology. For example, SD-OCT has demonstrated a Pearson correlation coefficient of r = 0.94 with histological retinal thickness measurements, confirming its accuracy in structural imaging [33]. Additionally, in oncology, OCT has shown sensitivity of ~85% and specificity above 90% in differentiating malignant from benign lymphatic tissue [34], and ~80% agreement in intraoperative tumor margin detection compared to histology in oral cancer [35].

However, OCT also has several limitations compared to histological examination: Limited Tissue Penetration: OCT typically penetrates only a few millimeters, making it less effective for deep tissue imaging [36]. Lower Cellular Resolution: While OCT provides high-resolution images at micrometer scale, it does not match the subcellular detail seen in histology [37]. Lack of Molecular Analysis: Histopathology allows for immunohistochemical staining and genetic profiling, whereas OCT primarily provides structural imaging [29].

### 2.2. OCT for Tissue Characterization and Guiding Biopsies

OCT has demonstrated its potential as a powerful tool for tissue characterization and biopsy guidance, significantly enhancing diagnostic accuracy and procedural efficiency across various medical fields. OCT enables precise biopsy targeting, reducing sampling errors and improving disease detection rates [28,38]. Traditional biopsy techniques often rely on blind sampling, where tissue is extracted based on preoperative imaging or physician estimation, which can result in false negatives or inadequate samples. In contrast, OCT-guided biopsy enables real-time visualization of tissue architecture, allowing clinicians to identify regions of interest (ROIs) and select optimal sampling sites, thereby improving diagnostic capability. Through improved accuracy of tumor identification and reduced likelihood of non-diagnostic samples, OCT represents a critical advancement in the field of diagnostic medicine [36,39].

One of the primary advantages of OCT in biopsy guidance is that it analyzes tissue layer disruptions, increased backscattering, and irregular tissue morphology. OCT provides a detailed map of suspicious regions, allowing for precise, targeted biopsy sampling [38,40]. This capability is particularly valuable in colorectal and esophageal cancer screening, where submucosal abnormalities can be detected with higher sensitivity than traditional white-light endoscopy [39].

Furthermore, OCT provides real-time intraoperative imaging, enabling surgeons and pathologists to assess tissue margins and tumor boundaries without needing frozen section analysis or extended pathology wait times [21,37]. This feature is particularly beneficial in breast-conserving surgery, where ensuring clear surgical margins is crucial in reducing recurrence rates [41]. OCT has also demonstrated success in urological surgeries, where cross-polarization OCT (CP-OCT) has been utilized for bladder cancer resection, providing feedback in real time on tissue characteristics and minimizing unnecessary excess tissue removal [42].

As research continues to advance, OCT-guided biopsy is expected to integrate with artificial intelligence (AI) and multimodal imaging to further refine tissue classification and automate suspicious region detection [43]. Thus, OCT-guided biopsy represents a significant advancement in precision diagnostics, improving sampling accuracy, reducing procedural errors, and ultimately enhancing patient outcomes [29].

### 2.3. Clinical Applications of OCT

In ophthalmology, OCT is primarily utilized for the detection and monitoring of macular diseases, including diabetic retinopathy, age-related macular degeneration (AMD), and retinal vein occlusion. Studies have shown that SS-OCT can effectively screen for macular pathologies in cataract surgery patients, with sensitivity and specificity rates ranging from 63 to 83% and 72 to 89%, respectively [44,45]. As previously highlighted, OCT plays a critical role in evaluating retinal integrity, assisting in the diagnosis of conditions such as macular edema and retinal vein occlusion [43,46].

In dermatology, OCT provides detailed imaging of vascular networks and tissue morphology, enhancing the diagnostic accuracy of non-melanoma skin cancers [15,16]. Additionally, dynamic OCT has been used to visualize microvascular changes in conditions such as psoriasis, port-wine stains, and chronic venous ulcers, aiding in disease monitoring and treatment evaluation [47,48].

In vascular imaging, intravascular OCT (IVOCT) has enabled the detailed visualization of atherosclerotic plaques, vascular occlusions, and neovascularization, making it an essential tool in cardiology and ophthalmology [21,49]. Its ability to provide quantitative measurements of vessel morphology has been particularly beneficial in assessing diabetic retinopathy and cardiovascular disease and guiding endovascular interventions [25].

Notably, the clinical utility of OCT in these domains has been further expanded by the emergence of portable and handheld OCT systems. These innovations offer significant benefits in settings where traditional equipment is impractical, such as rural clinics, bedside diagnostics, or outpatient care. Recent developments include low-cost handheld OCT platforms capable of producing clinically viable images with 8 µm resolution, and compact briefcase systems enhanced by real-time machine learning, particularly useful for otolaryngology applications [50]. The increasing commercial availability of optical components has further accelerated the development of high-functioning, cost-efficient portable OCTs [51]. While these systems are also making strides in non-medical fields like plant science [52], challenges remain in achieving parity with traditional OCT systems in terms of image quality and acquisition speed. New systems are now reaching 120 kHz A-scan rates, narrowing this performance gap [53]. Continued innovation and long-term validation are vital to ensuring sustained clinical performance and wider adoption.

## 3. OCT in Oncology: Tumor Markers, Personalized Medicine, and Real-Time Treatment

### 3.1. The Role of OCT in Tumor Detection and Tumor Microenvironment Analysis

OCT has been extensively studied across various cancers, with significant applications in the diagnosis and assessment of malignancies in the skin, ocular tissues, breast, lungs, cervix, endometrium, colorectal region, brain, bladder, prostate, ovary, and the gastrointestinal tract [54]. OCT enables the differentiation between benign and malignant ocular tumors by visualizing key features such as hyper-reflective epithelial thickening and abrupt transition zones (Figure 2) [55,56]. In breast cancer, OCT demonstrates over 90% sensitivity in detecting residual cancerous tissue, aids lesion characterization, and, through polarization-sensitive OCT (PS-OCT), effectively differentiates breast cancer subtypes by analyzing the additional information of collagen birefringence, which provides critical insights into tumor invasiveness and treatment response [57,58,59].

Furthermore, in lung cancer, studies have demonstrated that OCT can effectively identify malignant pulmonary nodules, with some research indicating that it can achieve diagnostic accuracies comparable to histopathological assessments [60,61]. In the detection and evaluation of cervical cancer, particularly for identifying cervical intraepithelial neoplasia (CIN) and other precancerous lesions, studies have revealed that OCT can achieve sensitivities ranging from 85% to 98% in detecting cervical lesions, although specificity can vary widely, often reported between 39% and 81% due to high false-positive rates [62,63,64]. Also, OCT has shown significant potential in the detection and evaluation of endometrial cancer; it is capable of differentiating normal, hyperplastic, and malignant tissues. Advanced OCT technologies, such as real-time rotational OCT, have been shown to effectively delineate endometrial layers with strong histological correlation [65]. Additionally, OCT’s ability to assess endometrial vascularization and neovascularization offers critical insights into physiological and pathological changes, supporting its application in infertility assessment and cancer detection [65,66].

Moreover, in colorectal cancer, OCT has been validated as an effective tool for detecting dysplasia in submucosal layers, a critical step in early diagnosis and intervention [39]. The use of full-field OCT (FF-OCT) in neurosurgery is currently being explored for its potential to enhance real-time visualization of brain tissues, aiding in the precise removal of tumors and improving post-surgical outcomes [67]. In bladder cancer, CP-OCT has shown 94% sensitivity and 84% specificity in detecting high-grade urothelial dysplasia and carcinoma, making it an important tool for guiding biopsies and tumor resections [42].

While the diagnostic value of OCT in prostate cancer detection remains debated, recent advancements in needle-based OCT systems have demonstrated its potential for in vivo imaging and real-time identification of malignant tissues, which could revolutionize instant diagnosis and treatment planning, ultimately improving patient outcomes [68,69]. Over and above that, OCT has shown great potential in detecting and characterizing ovarian cancer based on microstructural changes, including collagen alterations [70,71]. The integration of OCT with AI techniques, such as convolutional neural networks (CNNs) for image processing, has demonstrated promising results in automating cancer detection, improving diagnostic accuracy, and reducing pathologists’ workload [72]. Additionally, FF-OCT and intraoperative OCT applications, including their use in laparoscopic procedures, have enhanced real-time tumor margin assessment and metastasis detection [71]. Last but not least, in the oral cavity, OCT could identify dysplastic lesions and oral squamous cell carcinoma [35]. Also, in the esophagus, OCT is instrumental in detecting early neoplastic changes associated with conditions such as Barrett’s esophagus, which can lead to esophageal adenocarcinoma. OCT’s ability to enable high-resolution imaging of the esophageal mucosa assists in identifying dysplastic areas that may not be visible through conventional endoscopy [39].

### 3.2. OCT-Guided Personalized Cancer Treatment

OCT enables real-time imaging during surgical procedures, allowing for immediate visualization of tumor characteristics and surrounding tissues. This capability is particularly crucial in complex surgeries where accurate tumor delineation directly impacts surgical success and long-term patient outcomes. Intraoperative OCT has been extensively studied for its role in guiding tumor resection by providing instant feedback on margin status.

Researchers demonstrated the utility of OCT in monitoring microstructural changes in pancreatic tumor organoids treated with radiation [73]. By capturing these changes in real time, clinicians can make immediate adjustments to treatment protocols, enhancing the precision of cancer therapies [74]. Additionally, a study explored the integration of OCT with confocal microscopy, effectively creating a “virtual biopsy” that combines high-resolution imaging with deep tissue penetration [75]. This approach enables immediate evaluation of skin lesions and supports real-time treatment decisions during dermatological procedures [29].

The application of OCT in breast-conserving surgery is particularly promising. A multicenter study demonstrated that intraoperative OCT could be used to assess tumor margins, ensuring complete excision and reducing the likelihood of reoperation [76,77]. By allowing surgeons to scan excised tissue in real time, OCT significantly lowers the incidence of positive margins. Furthermore, OCT has been investigated for its role in lymph node assessment during oncologic surgeries [78]. Studies suggest that OCT can detect micrometastases that might be missed by conventional histopathology, thereby enhancing real-time staging and treatment planning. These applications reinforce the value of OCT in improving surgical precision and reducing recurrence rates [28,79].

### 3.3. Key Limitations of OCT in Oncology

Despite its advantages, several limitations must be addressed to maximize its clinical utility. One of the most significant limitations of OCT in oncology is its limited imaging depth, which typically ranges between 1 mm and 2 mm in biological tissues. This limitation arises due to the high scattering of near-infrared light in biological tissues, which limits the penetration of OCT signals beyond superficial layers. As a result, deeper tumors or infiltrative malignancies may not be adequately visualized, limiting the comprehensive assessment of tumor margins and depth of invasion [74]. Another challenge in OCT imaging for oncology is its limited specificity in differentiating between tissues. While OCT excels in identifying tissue microarchitecture, it may not always reliably distinguish dysplastic lesions from invasive cancers, leading to potential false positives or false negatives [29]. In a study by Sunny et al. [35], OCT exhibited high sensitivity in detecting dysplastic lesions, but its specificity varied significantly depending on tissue type and imaging parameters. Also, differences in tumor regions, such as highly proliferative vs. necrotic areas, can result in OCT capturing inconsistent tissue characteristics, making standardized assessment difficult [80].

Recent advancements in nanotechnology-based contrast agents have shown promise in overcoming many of OCT’s limitations in oncology. Consequently, there is an increasing need for contrast-enhanced imaging approaches to improve OCT’s specificity in oncological applications. Nanoparticle-enhanced OCT imaging integrates tumor-specific nanoparticles, allowing for targeted imaging of cancerous tissues with improved sensitivity and contrast. This approach leverages nanoparticles conjugated with biomolecular markers, enabling OCT to highlight malignant regions with greater specificity [79].

## 4. Nanoparticles in OCT Imaging: Enhancing Diagnostic and Therapeutic Capabilities

### 4.1. The Role of Nanoparticles in OCT Contrast Enhancement

Hence, as it has been mentioned in the previous section, nanoparticles have emerged as pivotal agents for enhancing the contrast, sensitivity, and optical scattering in OCT through several mechanisms rooted in their unique optical properties. The integration of nanoparticles into OCT technology has shown substantial promise in overcoming the inherent limitations of standard OCT, particularly concerning tissue optical contrast. One of the primary ways nanoparticles improve OCT is through their peculiar scattering properties. The effective manipulation of the scattering cross-section of nanoparticles has been well-documented [27,81]. For instance, it has been shown that the size and shape of gold nanoparticles (GNPs, also referred to as AuNPs) can be tuned to optimize their scattering characteristics, thus improving the quality and contrast of OCT images in challenging environments like highly scattering biological tissues [81,82,83]. Another noteworthy enhancement comes from the development of nanoparticles that serve as photothermal agents, which leverage the thermal response of the nanoparticles under laser excitation to alter the phase and the intensity of the OCT signal [84,85]. This adjustment helps to augment the imaging contrast further by increasing backscattering from the regions containing nanoparticles. Additionally, this method allows for deeper tissue imaging, which is particularly beneficial in complex structures such as tumors, where traditional imaging might falter due to scattering effects [84,86]. Enhanced imaging sensitivity has been demonstrated in vivo, where nanoparticles have markedly improved the visibility of microcirculatory dynamics in tumor environments and facilitated the identification of distinct pathological changes [84,87]. Furthermore, advancements in the design of nanoparticle contrast agents, including the use of Janus microspheres and multifunctional nanoparticles, have broadened the scope of OCT applications. These innovative materials are capable of enhancing both absorption and scattering concurrently, thus producing a synergistic effect that significantly boosts imaging resolution and depth penetration in various tissues, including in vivo scenarios [84,88].

Also, particularly when it comes to metallic nanoparticles such as gold and silver, a fundamental mechanism by which nanoparticles increase optical scattering is through plasmon resonance. Metallic nanoparticles can support localized surface plasmon resonances (LSPRs), which occur when the conduction electrons in the nanoparticles oscillate at specific frequencies in response to incident light. This phenomenon significantly amplifies the scattering cross-section of the nanoparticles, leading to enhanced backscattering of light in the OCT system. For instance, specific studies have reported that GNPs exhibit highly efficient scattering processes, where the scattering cross-section can reach up to 0.7 × 10^−10^ cm^2^ per nanoparticle [27]. Such strong scattering capabilities are particularly effective in the near-infrared region, commonly used in OCT imaging of biological tissues. Additionally, the control over the size and shape of nanoparticles allows for tuning their optical properties, resulting in varied scattering efficiencies [89]. The architecture of nanoparticle assemblies can also lead to synergistic increases in scattering, further enhancing overall imaging contrast [82]. Another important mechanism involves the enhancement of scattering due to interactions between nanoparticles and the surrounding medium. The relative refractive index contrast between nanoparticles and their environment plays a critical role in the backscattering of light. In biological media, nanoparticles can effectively increase the backscattering coefficient, allowing for clearer differentiation between tissue and lesions, and facilitating enhanced imaging capabilities [27,90]. Moreover, employing engineered nanoparticles with magnetic and plasmonic properties presents a new frontier in OCT image enhancement. These hybrid nanoparticles can increase scattering and dynamically adjust their properties in response to external magnetic fields, improving contrast and signal clarity during imaging procedures [91].

### 4.2. Types of Nanoparticles for OCT Applications

Multiple types of nanoparticles are under investigation as contrast agents in OCT due to their unique optical properties, which expand the capability of OCT for enhanced imaging in biomedical applications. Notably, AuNPs and silver nanoparticles (AgNPs) stand out prominently, with silicon nanoparticles (SiNPs) and various hybrid nanoparticle systems also showing potential. Gold nano bipyramids are extensively studied for OCT due to their strong light-scattering ability resulting from surface plasmon resonance and tunable absorption properties that can be optimized for specific imaging applications, especially in vivo settings [84,92]. Research indicates that these structures improve the delineation of vascular structures and help visualize intricate biological processes, broadening their applicability in areas like melanoma research [82,84]. AgNPs’ localized SPR provides strong scattering signals, which can significantly enhance contrast during imaging. Recent studies have demonstrated the application of AgNPs in SS-OCT, showing favorable results in improving the imagery of animal tissues and outperforming more conventional contrast agents such as titanium dioxide (TiO₂) nanoparticles. The manipulation of AgNPs’ size and shape further enhances capabilities, allowing for tailored imaging based on specific biological contexts [93].

SiNPs are gaining interest for their high refractive index and favorable scattering characteristics, making them suitable candidates for contrast enhancement in OCT [83,94]. Emerging materials, such as plasmonic copper sulfide nanoparticles, are being explored as innovative contrast agents that operate differently from traditional scattering agents. Rather than scattering light, copper sulfide nanoparticles absorb probing light, enabling improved contrast in OCT images by creating dark contrast properties at specific optical transparency windows [86]. Recent discoveries indicate that integrating magnetic and plasmonic materials can yield innovative contrast agents capable of optimizing OCT while assisting in molecular recognition tasks, thereby enhancing diagnostic capabilities in clinical practice [95,96]. This novel approach can significantly enhance visualization in applications requiring differential imaging techniques.

AuNPs enhance OCT due to their unique scattering properties, influenced by their size, shape, surface chemistry, and strong light-scattering capabilities [84,97]. Various forms of AuNPs, including nanoshells, nanorods, and nanoprisms, can be engineered to manipulate their scattering properties, tailoring them for specific OCT applications [89]. Furthermore, the ease of conjugation of gold nanoparticles with antibodies or peptides enhances their ability to target specific tissues or cells, significantly increasing the contrast and specificity of OCT imaging in pathological situations such as cancer [82]. Iron oxide nanoparticles, particularly superparamagnetic forms, exhibit strong light-scattering and magnetic properties that enhance their utility in imaging technologies, including OCT. These nanoparticles can alter the local magnetic field and induce localized susceptibility, resulting in increased contrast in OCT images. Their size allows for enhanced scattering efficiency, and they can be utilized in magnetomotive OCT applications to track cellular uptake and migration effectively [98]. This function is particularly valuable in characterizing inflammatory processes and studying cardiovascular diseases through the imaging of macrophage activity at vasculature sites [91]. Quantum dot nanoparticles (QDs) enhance OCT imaging through their unique optical properties, including size-tunable fluorescence and high quantum yield [99]. Quantum dots are particularly valuable because they can be engineered to emit at specific wavelengths that align with the imaging capabilities of OCT systems, thus providing improved contrast and imaging depth [100]. The tunability of their emission spectra allows for simultaneous imaging of multiple targets, facilitating comprehensive assessments of biological processes in real time [99].

Biocompatible nanoparticles have emerged as a crucial innovation in medical applications, offering enhanced drug delivery, improved imaging capabilities, and reduced systemic toxicity. One of their key advantages lies in their improved stability and biodegradability, which ensures controlled drug release and minimal adverse effects. Many biocompatible nanoparticles are designed to remain stable under physiological conditions, reducing the likelihood of premature drug release. Additionally, biodegradable nanoparticles, such as those composed of poly(lactic-co-glycolic acid) (PLGA) or chitosan, degrade into non-toxic metabolites over time, facilitating clearance from the body and minimizing long-term toxicity risks [101,102]. This property is particularly beneficial for sustained-release drug delivery systems and for reducing chronic exposure to potentially harmful materials. Another significant advantage of biocompatible nanoparticles is their functional versatility, which allows them to serve multiple roles simultaneously. These nanoparticles can be engineered to act as carriers for drug molecules, imaging agents, or therapeutic agents while also undergoing surface modifications to enhance targeting efficiency or improve drug-loading capacity. Such adaptability is particularly advantageous in combination therapies, where multiple treatment modalities can be integrated within a single nanoparticle system [103]. Additionally, biocompatible nanoparticles demonstrate reduced immunogenicity, meaning they are less likely to be detected and degraded by the immune system. This feature enables prolonged circulation in the bloodstream, which is crucial for therapies requiring sustained drug delivery to specific target sites without triggering an adverse immune response [104].

Despite their advantages, biocompatible nanoparticles present several challenges that must be addressed for widespread clinical use. One of the most significant hurdles is the complex production process and quality control required to ensure uniformity in size, shape, and surface chemistry. Variations in nanoparticle synthesis can lead to inconsistencies, posing challenges in scalability and clinical reproducibility [105,106]. Additionally, while biocompatibility minimizes toxicity risks, certain nanoparticles, such as silver-based nanoparticles, have raised concerns regarding cytotoxic effects. These nanoparticles may interfere with cellular pathways, leading to unintended biological consequences [106,107]. Another growing concern is the environmental impact of nanoparticle production and disposal. The long-term effects of nanoparticles on wildlife and ecosystems remain largely unexplored, necessitating rigorous environmental impact assessments to mitigate potential hazards [108]. Finally, challenges in clinical translation persist despite promising preclinical results. Factors such as nanoparticle stabilization during storage and transport, patient variability, and integration into existing therapeutic frameworks pose practical implementation challenges [104,109]. To overcome these barriers, ongoing research is focused on optimizing nanoparticle formulations, improving biocompatibility, and developing standardized protocols for regulatory approval. Table 1 summarizes all the benefits and difficulties around nanoparticle applications in OCT (Table 1).

### 4.3. Tumor-Targeting Nanoparticles in OCT Imaging for Personalized Medicine

OCT can be employed to monitor changes in the optical properties of tissues after administering nanoparticles, providing insights into their biodistribution and localization [92,110]. A recent study investigated the use of OCT in conjunction with microneedle-based transdermal drug delivery systems. This research highlighted how OCT’s chronological assessment capabilities can evaluate the penetration depth and distribution of nanoparticles administered via microneedles. Specifically, OCT allowed for the visualization of microchannels created by microneedling and the resulting drug delivery efficiency, thus demonstrating a dual role of monitoring both the device’s performance and the biodynamics of nanoparticle transport in vivo [111]. Moreover, certain studies have demonstrated that OCT can effectively monitor the deployment of nanoparticles in ocular applications [112,113,114]. Evidence shows the effectiveness of intraoperative OCT (iOCT) in evaluating drug deposition within the subretinal space during surgeries utilizing nanoparticle formulations [115]. This provides critical feedback regarding the placement and integrity of therapeutics within sensitive ocular environments. Additionally, OCT has been shown to visualize the interactions between nanoparticles and biological barriers, allowing researchers to study how morphology influences biodistribution data. Another promising avenue is the pursuit of OCT-enhanced imaging of AuNPs, which have distinct optical properties facilitating their detection via OCT techniques. Such applications suggest that OCT is applicable for real-time monitoring of theranostic nanoparticles and could serve as an in vivo diagnostic tool by enhancing contrast imaging capabilities when nanoparticles are utilized [92]. One study demonstrated the potential to observe physiological changes in the skin during the transdermal delivery of AuNPs, allowing for quantitative assessments of therapeutic penetration and localization [116]. Table 2 outlines the key benefits (e.g., enhanced imaging contrast, theranostic applications) and challenges (e.g., toxicity, regulatory hurdles) of using nanoparticles in OCT (Table 2).

## 5. Future Directions in OCT Imaging for Pathology and Oncology

### 5.1. Overcoming OCT Limitations with Nanotechnology

Nanoparticles have significant potential to enhance the differentiation of tumor subtypes by serving as versatile platforms for delivering diagnostic and therapeutic agents that can recognize unique molecular features in various cancers. Each tumor subtype exhibits a distinct set of biomarkers, often characterized by specific protein expressions or genetic profiles. A study discussing prognostic implications in small cell lung cancer (SCLC) illustrates that specific molecular subtypes correlate with distinct immune characteristics and treatment responses, underscoring the potential for nanoparticle-mediated identification based on varying biomarker expressions [117]. Furthermore, research in ovarian cancer indicates that different tumor-associated markers are enriched in subtype classifications, suggesting that nanoparticle systems can be tailored to isolate and visualize these specific markers effectively [118].

Moreover, engineered nanoparticles can serve as powerful tools in the characterization and differentiation of breast cancer subtypes. The investigation of high-forming binding protein 17 (FBP17) has shown its expression to be linked to differentiation among ductal carcinomas, suggesting a role for FBP17 in subtype classification [119]. By combining these differentiation markers with nanoparticles that can bind selectively to them, researchers may enhance the resolution of subtype differentiation in diagnostic settings. Next-generation nanoparticles, particularly those engineered for enhanced contrast in imaging technologies, including OCT, are expected to transform tumor imaging paradigms. Recent advances in material science have led to multifunctional nanoparticles that incorporate imaging contrast agents, allowing them to highlight tumor tissues in real time [120]. These advancements may include the use of nanoscale agents that are sensitive to specific optical frequencies and provide enhanced contrast by exploiting the optical scatter of tumor microenvironment characteristics. In OCT, nanoparticles can be designed to reflect OCT light more efficiently in the presence of tumor markers, creating a stronger contrast between healthy and malignant tissues. By encoding optical properties into the nanoparticles in correspondence with specific biochemical markers or cellular structures, researchers can achieve high-resolution imaging that reveals details about tumor subtype heterogeneity [121]. Engineered nanoparticles can also be modified to respond uniquely to tumor microenvironment cues, such as pH or enzyme activity, enhancing their specificity in imaging applications. This can lead to improved visualization of not only tumor size and shape but also subcellular characteristics relevant to subtype classification. By conjugating nanoparticles with ligands that selectively bind to proteins overexpressed in specific tumor types, such as HER2 in breast cancers or CD44 in certain bladder cancers, researchers can utilize the enhanced imaging capabilities of these systems to delineate subtype features with improved clarity [122].

### 5.2. Multimodal Imaging: Integrating OCT with MRI, PET, and Ultrasound

Combining OCT with other imaging modalities like ultrasound, MRI, and PET significantly enhances diagnostic accuracy by utilizing the unique advantages of each technology [123,124]. This integration allows for a more comprehensive assessment, leading to improved identification of diseases and better patient management.

The combination of OCT and MRI is particularly effective in diagnosing neurodegenerative diseases such as multiple sclerosis (MS) and Alzheimer’s disease. OCT provides high-resolution images of the retina, allowing for the assessment of retinal nerve fiber layer (RNFL) thickness, which has been identified as a potential biomarker associated with brain atrophy detected by MRI [125]. Research shows that changes in retinal structure observable through OCT correlate with cognitive decline and brain volume in Alzheimer’s patients, indicating that OCT can provide valuable information about underlying neurological conditions [125,126]. The integration of these technologies not only enhances the specificity of diagnoses but also aids in monitoring disease progression more accurately. In oncology, the combination of OCT and MRI can elucidate the characteristics of tumors, particularly in organs such as the breast or prostate. MRI provides detailed soft-tissue morphology, while OCT can delineate the microstructural features of tumors in real time, which is crucial for surgical planning and treatment strategies [127].

Merging OCT with positron emission tomography (PET) enhances diagnostic capabilities, particularly in cancer detection and monitoring. PET provides insights into the metabolic activity of tissues, while OCT excels in structural imaging [128]. The simultaneous use of these modalities allows for the identification of malignant lesions that may not be evident via PET alone. Integrative approaches using OCT alongside functional imaging from PET have shown improvements in diagnostic accuracy when investigating various malignancies. PET can identify hypermetabolic areas indicative of malignancy, while OCT clarifies lesion boundaries and provides additional histological information, yielding a superior overall diagnostic profile [129].

Ultrasound imaging combined with OCT offers distinct advantages, particularly in assessing superficial lesions. While ultrasound provides excellent spatial resolution and can evaluate depth, it is limited in providing detailed microstructural information about soft tissues. Conversely, OCT delivers high-resolution images of retinal and other tissues, allowing for the assessment of structural anomalies that ultrasound cannot effectively capture. In dermatological applications, the integration of OCT with ultrasound can enhance the evaluation of skin lesions, providing a comprehensive approach to diagnose conditions such as skin cancers [127].

Recent studies indicate that multimodal imaging approaches, such as the combination of OCT with MRI and PET, can lead to significant improvements in diagnostic accuracy across various patient populations [130]. In patients with uncertain diagnoses, the incorporation of additional imaging modalities often results in more definitive diagnostics; up to 31% improvement in diagnoses has been noted, showcasing the power of combined data [131]. Moreover, in instances of complex conditions such as multiple sclerosis, integrating OCT with MRI has demonstrated a greater capacity to predict disability outcomes than using MRI alone [132,133]. Furthermore, in conditions like glaucoma, OCT’s ability to measure retinal thickness and nerve fiber layer integrity complements ultrasound’s ability to assess optic disc morphology, leading to enhanced diagnostic reliability for glaucoma detection [134]. This combination can help ensure that patients receive prompt and appropriate interventions, ultimately improving prognoses.

While such integrated approaches have the potential to enhance diagnostic accuracy and provide more comprehensive clinical information, several obstacles inhibit their widespread implementation in clinical practice. One of the primary challenges in multimodal imaging with OCT arises from technical limitations associated with each imaging modality. For instance, OCT is highly sensitive to motion artifacts due to its reliance on coherent light, and any patient movement can lead to suboptimal images [135]. Secondly, combining data from multiple imaging modalities increases the volume of information that clinicians must manage. The integration process is often cumbersome, necessitating advanced software solutions and skilled personnel capable of interpreting the resulting multimodal data [130].

### 5.3. Personalized Medicine: Combination of AI and OCT-Guided Precision Oncology

The combination of smart probes, biosensors, and AI significantly enhances OCT-guided diagnostics, specifically in the realm of personalized medicine [136,137]. This integration provides a multifaceted approach that enables more accurate diagnostic assessments, tailored treatment strategies, and improved patient outcomes [128]. Firstly, by using targeted biosensors, clinicians can detect subtle biochemical changes related to various diseases. This is particularly valuable in oncological applications where early detection is crucial. Combining AI with data from these smart probes allows for sophisticated analysis and pattern recognition; this increases the probability of identifying cancerous tissues with greater accuracy compared to standard OCT imaging alone [138]. Secondly, coupled with AI, these probes can analyze data instantaneously, providing immediate feedback regarding the cancer microenvironment, metabolic changes, or inflammatory responses in tissues. For instance, AI algorithms can process OCT data to monitor tumor responses to treatment, allowing clinicians to make swift adjustments to personalized therapy based on live data [139]. This capability is particularly beneficial in managing chronic diseases where adjustments to treatment protocols are frequently needed.

Additionally, the substantial amount of data generated by OCT systems integrated with biosensors can be efficiently managed by AI algorithms. Traditional methods of data interpretation can be time-consuming and prone to human error. AI technology automates the diagnostic process by analyzing patterns in OCT scans combined with biosensor outputs, reducing variability and improving diagnostic reliability [140]. Recent studies have demonstrated that AI can analyze OCT images of oral lesions with a sensitivity and specificity of over 98%, assisting clinicians in making informed decisions [141]. Such automated systems fulfill a crucial role in personalized medicine by providing consistent monitoring across patient populations. Next, the integration of smart probes and AI facilitates the development of personalized treatment plans tailored to the patient’s unique biological profile. For example, biosensors that detect specific biomarkers allow clinicians to evaluate a patient’s response to therapy in real time. With continuous data acquisition, AI can correlate biomarkers with treatment outcomes, optimizing personalized therapeutic approaches for individual patients [142]. This precision medicine approach significantly enhances treatment effectiveness while minimizing potential side effects by allowing for timely adjustments based on real-time data.

Moreover, smart probes and biosensors can be miniaturized and designed for point-of-care use, making advanced diagnostics accessible outside traditional clinical settings. This portability enables earlier interventions and monitoring in rural or underserved areas, contributing to timely personalized medicine strategies [143]. For instance, in oral oncology, portable biosensors paired with AI-driven OCT diagnostics allow for non-invasive assessments in outpatient settings, facilitating earlier diagnoses and interventions for at-risk populations [144].

In addition, recent studies have demonstrated the utility of advanced AI architectures, such as EfficientNetV2 and ConvNeXt, in identifying subtle biomarkers from OCT images, achieving high accuracy in applications like diabetic macular edema and coronary artery plaque detection [139,145]. Importantly, AI algorithms offer an objective assessment of structural anomalies, as demonstrated by Daxenberger et al. in actinic keratosis analysis using LC-OCT, further reducing subjectivity in clinical evaluations [146]. These findings reinforce the growing reliability of AI in supporting real-time, high-accuracy image interpretation across diverse clinical contexts.

While AI integration with OCT shows strong potential, several limitations persist. These include challenges with data quality and variability [147], imaging artifacts such as blurriness and poor contrast [135], and OCT’s limited imaging depth of 2–3 mm, which restricts its use for deeper tissues [148]. Moreover, the interpretability of AI outputs remains a concern, as many systems function as “black boxes” and may reduce clinician confidence [149]. Ethical issues, including data privacy and algorithmic bias, also need to be addressed for broader clinical adoption [150]. Table 3 summarizes AI applications in OCT, including techniques, clinical contexts, and reported limitations.

## 6. Conclusions

OCT has proved to be a transformative imaging technology, providing high-resolution, real-time visualization of tissue microstructures in various pathological conditions. In oncology, OCT facilitates early tumor detection, intraoperative margin assessment, and real-time monitoring of treatment response, offering a non-invasive and precise alternative to conventional imaging modalities. However, despite its advantages, OCT faces limitations, particularly in imaging depth and specificity, which restrict its ability to assess deep-seated tumors and differentiate between malignant and benign lesions with high accuracy. The integration of nanoparticles into OCT imaging has been proposed as a promising solution to enhance contrast, improve tumor delineation, and enable targeted imaging of specific biomarkers. Also, to address OCT’s limited imaging depth in certain dense tissues, hybrid approaches using longer-wavelength sources (e.g., 1300 nm) and adaptive optics are under development [151,152]. While primarily engineering-driven, these advances hold promise for extending OCT’s clinical utility in deep-seated tumor visualization and should be considered in future translational studies.

Nanoparticles, particularly gold and superparamagnetic iron oxide nanoparticles, have demonstrated significant potential in overcoming OCT’s limitations by improving scattering properties, increasing imaging contrast, and enabling functional imaging at a molecular level. Their ability to serve as contrast agents and therapeutic carriers underscores their role in advancing OCT-based precision oncology. Furthermore, the combination of OCT with multimodal imaging techniques such as MRI, PET, and ultrasound has shown promise in enhancing diagnostic accuracy by leveraging complementary imaging strengths.

Beyond oncology, OCT’s integration with artificial intelligence (AI) and biosensors has further strengthened its role in personalized medicine. AI-driven image analysis facilitates real-time tumor characterization, aiding in the optimization of treatment strategies and minimizing unnecessary tissue removal during surgery. The incorporation of smart and stimuli-responsive imaging agents enables OCT to provide dynamic insights into tumor microenvironment changes, opening new possibilities for targeted therapy and theranostics.

Despite these advancements, challenges remain in translating nanoparticle-enhanced OCT and multimodal imaging into routine clinical practice. Issues related to biocompatibility, regulatory approval, and scalability must be addressed to ensure safe and effective implementation. Future research should focus on refining nanoparticle formulations, enhancing targeting specificity, and integrating AI-driven image processing to optimize OCT’s clinical utility. By addressing these challenges, OCT has the potential to become an indispensable tool in precision oncology, facilitating early diagnosis, real-time treatment monitoring, and personalized therapeutic interventions, ultimately improving patient outcomes.

## Figures and Tables

**Table 1:** Comparison of nanoparticle types for OCT imaging (summarizing properties, advantages, and challenges).

Nanoparticle Type	Optical Property	Application in OCT	Advantages	Challenges
AuNPs	SPR	Contrast Enhancement	High Biocompatibility	Cost, Aggregation Issues
AgNPs	Strong Scattering	Image Enhancement	High Stability	Toxicity Concerns
SiNPs	High Reflective Index	Deep Tissue Imaging	Biodegradable	Limited Studies
QDs	Fluorescence	Multiplex Imaging	Tunable Emission	Potential Cytotoxicity

**Table 2:** Advantages and challenges of nanoparticles in OCT-based personalized medicine.

Advantage	Description	Example Nanoparticles
High-Contrast Imaging	Improves visualization of microstructures	AuNPs, AgNPs and QDs
Theranostic Capabilities	Enables simultaneous diagnosis and treatment	Magnetic, Hybrid Nanoparticles
Real-Time Monitoring	Facilitates intraoperative tracking	Plasmonic Nanoparticles
Biodegradability	Minimizes long-term toxicity	PLGA, Chitosan

**Table 3:** Summary of recent AI applications in OCT across clinical contexts.

Clinical Application	AI Model(s) Used	Imaging Modality	Dataset Characteristics	Reported Outcome	Limitation
Diabetic Macular Edema Detection	EfficientNetV2, ConvNeXT	Retinal OCT	Custom-labled dataset, high-resolution OCT scans	High classification accuracy for edema biomarkers	Requires extensive training data; risk of overfitting on rare subtypes
Coronary Plaques Analysis	CNN-based model	Intravascular OCT	Small dataset (n < 300); manually labeled vulnerable plaques	88.46% plaque detection accuracy	Limited generalizability; needs external validation
Lesions Differentiation	Deep CNN	Retinal OCT	Oral lesion dataset; biopsy-verified classes	>90% accuracy in lesion classification	Model interpretability and real-time clinical integration
Actinic Keratosis Evaluation	Automated ML pipeline	LC-OCT	Clinical image set with dermatological annotation	Increased diagnostic accuracy vs. visual assessment	Artifacts and variability in LC-OCT images
Coronary Disease Classification	Ensemble CNN with multimodal fusion	OCT + Angiography	OCT paired with angiographic images	Enhanced specificity in arterial disease detection	High computational cost for real-time screening
